# Recent Advances on Jamming and Spoofing Detection in GNSS

**DOI:** 10.3390/s24134210

**Published:** 2024-06-28

**Authors:** Katarina Radoš, Marta Brkić, Dinko Begušić

**Affiliations:** 1 Faculty of Electrical Engineering, Mechanical Engineering and Naval Architecture (FESB), University of Split, 21000 Split, Croatia; krados@fesb.hr; 2 Ericsson Nikola Tesla d.d., 10000 Zagreb, Croatia; marta.balic@ericssonnikolatesla.com

**Keywords:** GNSS, spoofing, jamming, detection methods, machine learning models, software-defined radio

## Abstract

Increased interest in the development and integration of navigation and positioning services into a wide range of receivers makes them susceptible to a variety of security attacks such as Global Navigation Satellite Systems (GNSS) jamming and spoofing attacks. The availability of low-cost devices including software-defined radios (SDRs) provides a wide accessibility of affordable platforms that can be used to perform these attacks. Early detection of jamming and spoofing interferences is essential for mitigation and avoidance of service degradation. For these reasons, the development of efficient detection methods has become an important research topic and a number of effective methods has been reported in the literature. This survey offers the reader a comprehensive and systematic review of methods for detection of GNSS jamming and spoofing interferences. The categorization and classification of selected methods according to specific parameters and features is provided with a focus on recent advances in the field. Although many different detection methods have been reported, significant research efforts toward developing new and more efficient methods remain ongoing. These efforts are driven by the rapid development and increased number of attacks that pose high-security risks. The presented review of GNSS jamming and spoofing detection methods may be used for the selection of the most appropriate solution for specific purposes and constraints and also to provide a reference for future research.

## 1. Introduction

It is almost impossible to imagine navigating modern life without the use of Global Navigation Satellite Systems. Applications for navigation and positioning have become unavoidable, whether we are traveling somewhere, looking for something, or doing our job as bus, truck, taxi, ship, or plane crew. Stable and precise synchronization is of key importance in mobile networks for the successful connection of base stations and real-time data transmission, as well as for navigation and positioning services. Mobile networks must be synchronized so that base stations whose coverage overlaps do not interfere with each other causing call drops or service degradation. Due to the loss of synchronization, there is a deterioration in the quality of mobile transmission, a drop in the number of successful calls, and a decrease in the number of users. One of the important sources of reference signals for synchronization and provision of navigation and positioning services is GNSS. Constant improvement of existing systems ensures better precision. However, due to the increasing use of satellite navigation systems, there are an increasing number of threats and risks such as malicious attacks targeting these systems [1].

In this review paper, we consider jamming and spoofing attacks in GNSS. Before the arrival of recent advances in communication and information technologies, the generation of such interferences required sophisticated knowledge and equipment. However, today these attacks can be carried out by almost anyone due to cheap equipment and the availability of various instructions. The most commonly used device for performing these attacks is a cheap software-defined radio (e.g., Universal Software Radio Peripheral (USRP), HackRF One, LimeSDR). Jamming is the deliberate interference, caused by emissions intended to render unintelligible or falsify the whole or part of a wanted signal according to the International Organization for Standardization (ISO) [2]. It is the transmission of a high radio frequency signal that is equal to or close to the frequencies at which GNSS receivers operate. According to the European Telecommunications Standards Institute (ETSI) TS 103 246-5 V1.3.1 (October 2020) specification [3], a spoofing attack is the transmission of signals intended to deceive location or timing processing into reporting false location or timing target data. The impact of a spoofing attack on a GNSS receiver is reflected in taking over the navigation system and spoofing the location of the receiver.

Navigation applications mostly rely on GNSS systems rendering them even more sensitive to threats [4]. Navigation systems are exposed to security risks in the face of spoofing attacks [5] and consequences in people’s lives. The GNSS community did not pay enough attention to these threats in the open literature until Humphreys et al. [6] developed a system for performing spoofing attacks. The spoofing attack is successfully performed and tested on a commercial standard receiver. The strategy for detecting spoofing attacks on cryptographically protected GNSS signals is presented in [7,8].

This paper gives a brief overview of, to the authors’ best knowledge, state of the art jamming and spoofing detection methods. In comparison with other similar papers, we also provide an overview of jamming detection methods and jamming and spoofing combination detection methods researched in the last few years. Potential detection methods include signal processing methods [9], data bit methods, position methods, and machine and deep learning (DL) methods recently.

Considering the available works, it can be concluded that in the last few years, the most popular methods for the detection of GNSS jamming and spoofing attacks are machine learning (ML) methods. The performances of a few different machine learning methods are compared in [10]. Their results show that classification and regression decision tree models have the best performance for the detection and classification of Global Positioning System (GPS) spoofing attacks when compared with other supervised and unsupervised models. In [11], the authors compare a few ML methods and show that Support Vector Machine (SVM) gives the best GPS spoofing detection results. On the other hand, authors in [12] also make a comparison of a few ML methods and show that K-Nearest Neighbors (KNN) gives better results in comparison to SVM. Authors in [13,14] agree with Shafique et al. [11] that SVM is a promising approach for spoofed signal detection. Chen et al. show that their multiparameter spoofing detection method on the dataset which is the combination of publicly available Texas Spoofing Test Battery (TEXBAT) and Oak Ridge Spoofing and Interference Test Battery (OAKBAT) datasets, gives a significant improvement compared to traditional methods. Most of the spoofing detection algorithms use a single publicly available dataset. Some publicly available datasets with spoofing scenarios are also used in the papers for ML methods training and testing as well as datasets created with simulators [14,15,16]. The application of Neural Networks (NN) is also often used, as demonstrated in previous studies [17,18,19].

Besides machine and deep learning methods, there is also detection by using Time of Arrival (ToA) [20], Direction of Arrival (DoA) [21], and National Marine Electronics Association (NMEA) messages analysis [22] which belong to data bit methods. There are signal processing methods where correlation peak monitoring [23], power-based methods [24] and antenna-based methods [25] belong. The method with pseudoranges measurements based on integrity check among different pseudorange measurements is described in [26,27]. Radio Frequency Fingerprinting (RFF) methods are widely used in the context of the Internet of Things, Wi-Fi, and cellular networks but not in the context of GNSS. Authors in [28,29,30,31] use RFF methods for detection of spoofed signals.

Many of these methods are also used to detect GNSS jamming signals, but also in situations where jamming and spoofing attacks are employed in combination.

This paper is organized as follows. Section 1 gives an introduction and related works. Jamming and spoofing principles are described in Section 2. In Section 3, spoofing detection methods are presented, and jamming detection methods in combination with spoofing detection methods are presented in Section 4. Finally, Section 5 concludes the paper.

## 2. GNSS Jamming and Spoofing Principles

**Figure 1:**
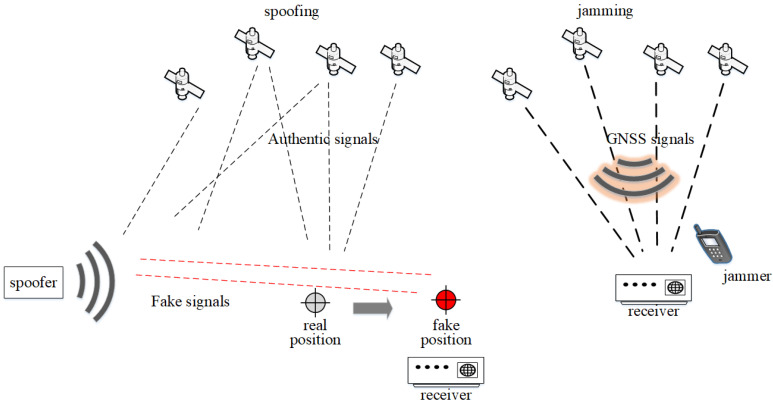
Main principle of spoofing and jamming attack.

The main principles of jamming and spoofing attack are shown in Figure 1. The spoofer (left) transmits fake signals, which are similar to authentic signals to the receiver. Fake signals are transmitted at higher power than authentic ones in order to trick the receiver into picking up those signals and subsequently begin tracking the fake satellites. The outcome of this attack is taking over the receiver’s navigation system and falsifying its location. In the case of a jamming attack (right), the jammer interferes with the authentic satellite signals and prevents the receiver from collecting and tracking authentic signals by transmitting the signals at the frequency near L1, L2, and L5 bands.

### 2.1. Jamming Attack

Jamming is an intentional transmission of a high-power radio frequency signal equal to or very close to the frequency of the device whose operation is to be prevented. It aims to prevent the receiver from collecting and tracking GNSS signals and navigating using GNSS signals. Jamming occurs due to the transmission of high-power radio frequencies near the L1, L2, and L5 frequency bands on which GNSS receivers operate. Jamming frequencies are intended to overload the receivers to the point that the receivers lose lock on the satellites, and have the main effect of rendering the GNSS system ineffective or degraded for users in the jammed area [32]. Because many devices transmit on frequencies close to GNSS receivers, it is possible that some of these devices unintentionally interfere with GNSS signals. Jamming is even more problematic than spoofing because GPS jammers are relatively much simpler in comparison to spoofing devices. In addition, they are easier to make than spoofing devices. Even small jammers that fit in the palm of a hand can have a range of several meters. A jammer can block all radio communications on any device operating on radio frequencies within its range and emit radio frequency waves that prevent the target device from establishing or maintaining the connection. Spoofing is more complex since the fake signal’s structure should be imitated and similar to the authentic one [5].

Authors in [33] classify jamming as follows:

1. Suppression jamming—the satellite navigation signal is suppressed by transmitting a jamming signal that has high power in the frequency band of the satellite navigation signal. In addition, the signal-to-noise ratio of the receiver is reduced and the satellite navigation signal is annihilated with the jamming signal. As a result, receiver positioning accuracy is reduced or unable to work properly. There are three types of suppression jamming:
  - Single-frequency jamming is the simplest jamming type in suppression jamming.
    (1) $$J\left(t\right)=Acos\left(2\pi f_{c}t\right)$$
    where J(t) is jamming signal, *A* is the amplitude of the single-frequency jamming signal, and $f_{c}$ is the carrier frequency of jamming signal.
  - Pulse jamming is the type in which a jamming signal is composed of continuous ideal rectangular pulses.
    (2) $$J\left(t\right)=Acos\left(2\pi f_{c}t\right)s\left(t\right)$$
    where *A* is the amplitude of the pulse jamming signal, $f_{c}$ is the carrier frequency of the satellite navigation signal, and s(t) is the ideal rectangular wave signal.
  - Sweep jamming has a similar form as single-frequency jamming. The difference is that the carrier frequency of sweep jamming changes with time, while the carrier frequency of single-frequency jamming is fixed.
    (3) $$J\left(t\right)=Acos(2\pi \left(f_{c}+f_{sweep}t\right)t)$$
    where *A* is the amplitude of the sweep jamming signal, $f_{c}$ is the carrier frequency of the satellite navigation signal, and $f_{sweep}$ is the sweep frequency.
2. Deception jamming works in a way that the source generates a deception signal which is similar to the real satellite navigation signal. The deception signal has a power slightly higher than the real signal. There is one more way to perform such an attack: the source repeats the real satellite or navigation signal in space. With this attack, the attacker achieves that the receiver “picks up” wrong information about the time and location and sends it as such.
  There are two types of deception jamming:
  - Generated deception jamming is a jamming method in which the attacker generates and transmits the deception signal. The generated signal has the same structure as the real navigation signal. It gradually replaces the real signal in the tracking loop under the signal control strategy and power advantage. After that, it controls the tracking loop to achieve the purpose of deception.
  - Repeater deception jamming adds a certain time delay based on receiving the real satellite navigation signal. After that, it repeats the signal through power adjustment to make the satellite navigation receiver receive the repeater signal, and thus a false signal is transmitted.

### 2.2. Spoofing Attack

GNSS spoofing attack refers to the intentional transmission of fake GNSS signals to deceive the receiver to misinterpret fake signals as authentic ones, and to falsify the receiver’s location. The basic tasks of GNSS receivers are to receive and separate signals from satellites, calculate pseudoranges for each satellite based on signal reception time, demodulate the navigation message to obtain ephemeris data, and estimate the Position, Velocity, and Time (PVT) solution. In [34,35], the authors show that it is easy to spoof smartphone locations using a simplistic spoofing attack. The proposed approach is simple and economical because the spoofing attack is performed using a low-cost SDR (HackRF One) and an open simulator GPS-SDR-SIM.

In general, received GNSS signals can be described mathematically as a combination of several signals [36]

(4) $$y\left(t\right)=Re\{\sum_{i=1}^{N}A_{i}D_{i}\left[t-\tau_{i}\left(t\right)\right]C_{i}\left[t-\tau_{i}\left(t\right)\right]e^{j[\omega_{c}t-\phi_{i}\left(t\right)]}\},\;i=1,\ldots ,N$$

where *N* is the number of signals constituting the spreading code, $A_{i}$ is the amplitude of the *i*-th signal, $D_{i}$ is the data bit stream (data bits of each open-service GNSS signal) of the *i*-th signal, $C_{i}$ is *i*-th signal’s spreading code—a sequence of pulses that GNSS uses to spread the spectrum of the transmitted signal (mostly Binary Phase Shift Keying Pseudo-Random Noise Code—BPSK PRN code), $\tau_{i}\left(t\right)$ is the *i*-th signal’s code phase, $\omega_{c}$ is the nominal carrier frequency, and $\phi_{i}\left(t\right)$ is the beat carrier phase of the *i*-th signal. Spreading code $C_{i}$ is transmitted as part of the navigation message and allows the receiver to identify the satellite from which he received the signal.

Spoofer sends similar fake signals and replicates the carrier, PRN code, and data bits of each open-service GNSS signal, and the fake signal can be shown as follows

(5) $$y_{s}\left(t\right)=Re\{\sum_{i=1}^{N_{s}}A_{si}\hat{D_{i}}\left[t-\tau_{si}\left(t\right)\right]C_{i}\left[t-\tau_{si}\left(t\right)\right]e^{j[\omega_{c}t-\phi_{si}\left(t\right)]}\},\;i=1,\ldots ,N_{s}$$

where $\tau_{si}\left(t\right)$, $\phi_{si}\left(t\right)$, $A_{si}$ are code phase (compares the PRN codes to detect the distance between the satellite and the receiver), carrier phase (the phase of the carrier at the receiver), and amplitude of fake signals. Their values depend on the type of attack and differ from the authentic signal values. $N_{s}=N$ is the number of fake signals that is equal to the number of authentic signals. Each fake signal should have the same spreading/PRN code as the corresponding authentic signal to deceive the receiver and perform a successful spoofing attack, and usually, it broadcasts its best estimate of the same data $\hat{D_{i}}$ [36].

The total received signal during a spoofing attack is equal to

(6) $$y_{tot(t)}=y\left(t\right)+y_{s}\left(t\right)+v\left(t\right)$$

where v(t) is the received noise, which can be classified as internal and external to the receiver. External sources of noise are received by the antenna and include atmospheric noise, cosmic noise, human-made noise, and interference noise created from other users in an adjacent channel or in the same channel (it may be source of noise received from the transmitter (spoofer)). Internal noise is generated by components inside the receiver. This noise is the result of random processes such as the flow of charges in a device, or at a more fundamental level, the thermal vibrations in any component at a temperature above absolute zero. Radio receivers are made of components that generate noise. All components, passive (such as resistors), or active (transistor-based circuits) generate noise. The noise in active components actually limits the useful operating range of the device. Sources of white noise in GNSS receivers are usually characterized by receiver and antenna temperature noise. The antenna temperature models the noise entering the antenna from the sky as previously explained, while the receiver temperature models the thermal noise due to charge movement within the device such as the front end of the receiver. The receiver front end comprises the antenna, amplification, filtering, mixing, and conversion to quantized samples needed to convert received electromagnetic waves at one or more carrier frequencies into digitized waveforms at baseband or an intermediate frequency for signal processing. Additional noise occurs during the signal propagation from the antenna to the receiver as the noise of an active (amplifier) or passive (cable) component.

There are three different types of spoofing attacks.

**Figure 2:**
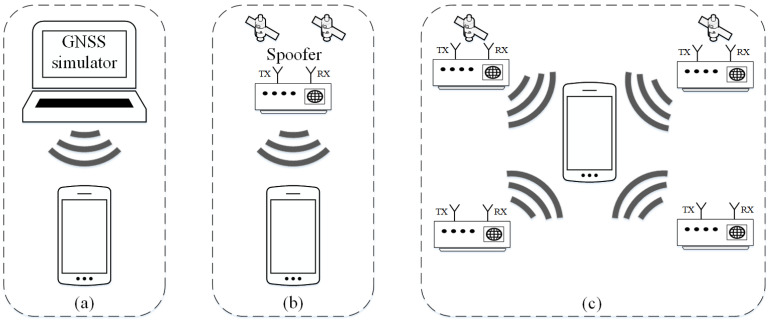
Types of spoofing attack: (**a**) simplistic, (**b**) intermediate, (**c**) sophisticated [37].

A simplistic spoofing attack is shown in Figure 2a. This attack is based on using a GNSS signal simulator to create a fake signal and transmit it to fool the receiver. This type of attack is very easy to implement because cheap equipment is used. A simplistic attack is easy to detect, considering that a high strength of the fake signal is needed for the receiver to ignore the authentic satellite signal and take the fake one, and the fake signal is not synchronized with the satellite constellation. Typically, these attacks are performed by first jamming the authentic GNSS signal to force the receiver to re-acquire and lock onto the fake signal. The result of a simplified attack is mostly jumps in PVT calculations [37].

An intermediate spoofing attack or receiver-based attack is shown in Figure 2b. In this type of attack, the spoofer (receiver/transmitter) has a built-in receiver that monitors and collects the parameters of the authentic satellite signal to generate the fake signal following the certain authentic signal and transmit it to the target receiver. This type of attack is complex because fake signals need to be synchronized with authentic signals. The feasibility of this attack has been proven as well as the possibility of changing the position of the receiver without lifting warnings or creating the discontinuities in the PVT solution [6].

A sophisticated spoofing attack is the most complex type of attack shown in Figure 2c. This type of attack uses several middle-level attackers that generate and transmit fake GNSS signals [37]. In this case, the attack cannot be easily detected by looking at the angle of arrival of the signal because the signals come from different angles and different attackers. These attacks have a much higher level of complexity due to the synchronization and communication process between each transmitter, which makes it very difficult to implement and unsuitable for real-time scenarios. Also, a sophisticated spoofing attack is not profitable from the economic point of view, because it requires additional and expensive equipment (several attackers, i.e., transmitters and antennas) [36].

## 3. Spoofing Detection Methods

GNSS signal spoofing detection methods have the primary goal of detecting spoofing attacks to alert the receiver that its location and time data are not correct. It is necessary to understand the characteristics of different attacks to develop a good defense against the attack itself. There are different methods of fake signal detection:

- methods based on observation of Carrier-to-Noise Ratio ($C/N_{0}$),
- pseudoranges, signal correlation functions,
- methods based on hardware simulator (e.g., simulator like Spirent) which are not economical [38],
- methods based on the use of an array of antennas,
- methods using NMEA messages [22],
- machine and deep learning methods.

A user device that receives fake signals and believes they are authentic can trigger dangerous behavior due to incorrect location or timing corrections. An example of this attack is shown in [4], where GPS signal spoofing is used to misdirect a drone into an unplanned dive and to divert a yacht off course. Therefore, spoofing defenses focus on detecting the attack to alert the attacked receiver that its calculated position and clock offset are unreliable. Table 1 shows the categories of spoofing detection methods in this review paper. A detailed categorization of spoofing detection methods is found below.

### 3.1. Signal Processing Methods

Signal processing methods include correlation peak monitoring, power-based, and antenna array processing methods.

#### 3.1.1. Correlation Peak Monitoring

**Figure 3:**
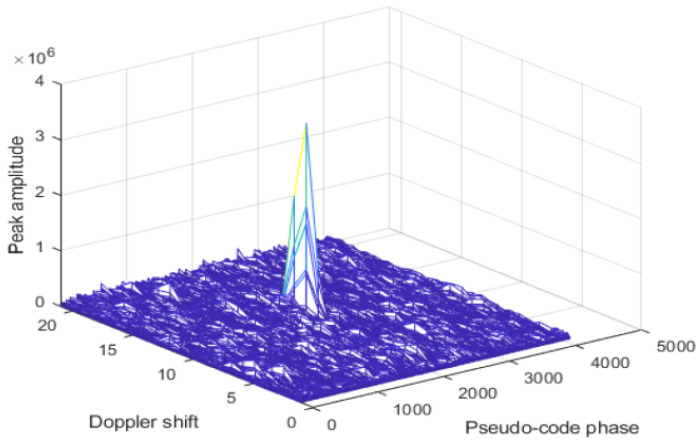
Real satellite signal in the capture phase [23].

**Figure 4:**
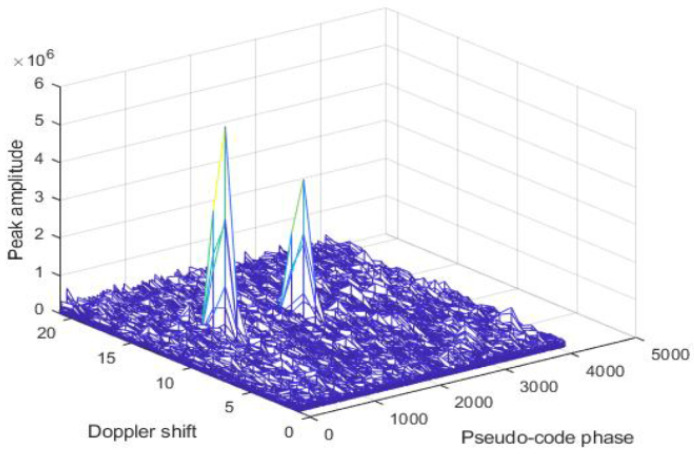
A spoofed signal exists in the capture phase with a delay of 100 chips [23].

Method for GNSS signals spoofing detection based on correlation peaks SQM and phase difference between fake and authentic signal is used in [23,39,40]. The paper [23] is focused on spoofing detection with low delay using the KNN machine learning method. Detection of the number of signal peaks is a key step for spoofed signal detection. The detection is based on fake signal detection by estimating the number of peaks that exceed a preset threshold when the receiver catches the signal. If there is only an authentic GNSS signal in the received signal, the value of only one correlation peak will exceed the preset threshold as shown in Figure 3. When there are spoofed signals, then there are two or more correlation peaks that are greater than the set threshold (Figure 3) and this method of detecting spoofed signals is valid when the phase difference between the fake signal and the authentic signal is large, i.e., larger than two chips. When the phase difference between real and spoofed signal is, for example, one chip, which is the case in Figure 4, the number of peaks is still one, so it is difficult to detect fake signals. The experimental results carried out in this paper showed that the proposed algorithm can detect spoofed signals with a delay greater than 0.6 chips and that has high accuracy. The authors in [39] show that the generative adversarial network (GAN) can reach more than 98% accuracy when the phase difference between the fake and authentic signal exceeds 0.5 chips and can be applied to situations where the fake signal is highly synchronized with the authentic signal. Authors in [40] also present a spoofing detection method based on correlation peaks. They propose a method which is based on spoofing correlation peak cancellation (SCPC). Their solution works by estimating the spoofing signal from the baseband sampling sequence and generating an inverse cancellation sequence, thereby countering the spoofing attack. The paper [41] presents a new metric developed by the authors due to limitations of SQM—its detection performances are limited when it is needed to detect spoofed signals which are heavily overpowered and with significant code phase shifts. With the help of the TEXBAT dataset, the effectiveness of the proposed detector is verified.

Since traditional SQM methods lack low detection accuracy and poor robustness, authors in [42] present a novel enhanced SQM method based on the application of the statistical Kolmogorov–Smirnov (KS) test. KS test monitors the correlator’s output of the GNSS receiver to identify the distortions in the correlator’s function. This method is tested on TEXBAT scenarios and the results show the accuracy improvement in the spoofing detection under the different power advantages. Furthermore, it can monitor the power variations caused by spoofing attacks, provides great detection sensitivity and robustness under different spoofing attack modes, and has low computational complexity.

Another enhanced approach for spoofing detection based on abnormal quadrature (Q) channel energy of correlators, which uses the estimation of the noise level as a parameter is proposed in [43]. Compared to the traditional SQM metrics, the Q energy detector has an overall detection ratio improvement by at least 20% when the $C/N_{0}$ exceeds 32 dB-Hz. This approach is also tested on all TEXBAT scenarios and it outperforms the traditional SQM in all of them, especially in overpowered scenarios and dynamic scenarios. The results show that increasing the power of the fake signal improves the relative detection performance of the Q energy detector compared to other SQM detectors, and it can be an effective spoofing detection method without the need to modify baseband correlators.

The authors in [44] focus on the classification of GNSS signals and classify them into classes: authentic, multipath, spoofed or jammed. The features they use to classify signals are average power and correlation distortion. Different machine learning methods were tested using an accuracy test and confusion matrix. Spoofed and jammed signals are easily distinguished from authentic signals due to their high average power and high degree of correlation distortion. Therefore, in the case of intentional disturbances (interference), this classification method is a powerful tool for navigation applications that use a GNSS receiver.

#### 3.1.2. Power-Based Methods

Signal strength monitoring is the simplest way to detect spoofing attacks because the strength of a fake signal is much higher than the strength of the authentic signal. A particular class of spoofing attack is meaconing. According to ETSI [3] TS 103 246-5 V1.1.1 (January 2016) specification, meaconing is the interception and rebroadcast of the navigation signals, typically with power higher than the authentic signal, to falsify positioning. Meaconing records the authentic GNSS signals and replays the signals through a transmitter with enough gain to overwhelm the authentic signal at the victim’s antenna [36]. In addition to the higher signal strength, the fake signal can be detected by the constant Doppler shift because the attacker is in the same location. In real satellite signals, the Doppler shift is dynamic and constantly changes depending on movement towards or away from the satellite. Additional parameters by which fake signals can be recognized are a constant pseudo-distance and a constant elevation angle because the attacker is transmitting from a fixed location. In the case of a dynamic attack, there should be several locations from which the attacker transmits and then it would be more difficult to detect fake signals. A traditional spoofed signal detection based on $C/N_{0}$ is proposed in [24], where the measured $C/N_{0}$ of received GNSS signals is compared to a known or expected value. The authors show that using the absolute power tracking technique significantly reduces the vulnerability area of the receiver in comparison to techniques that track C/$N_{0}$. The signal strength of GNSS signals is subject to physical degradation in unintentional (natural changes) or intentional ways [45]. In [67], in addition to $C/N_{0}$ for the detection of spoofing attacks, the authors also monitor pseudoranges. On the other hand, the authors in [48] consider the correlation distortion function together with pseudoranges and signal strength. Authors in the paper [68] use a combination of several methods—they focus on spoofing detection based on $C/N_{0}$ measurements, but after the jamming attack is prevented with the help of the antenna array.

A simplistic spoofing attack is carried out in [69] using software-defined radio. GPS signals are collected and replayed on smartphones. The GPS Test application is used for the tracking the results of the attack. The GPS Test application is used to monitor the attack results, i.e., parameters: available satellites and their $C/N_{0}$. In cases where $C/N_{0}$ discrimination has limited effectiveness, the receiver can measure the absolute strength of the correlation peaks, so this method is effective for detecting and discriminating attack sources. The authors in [46] show that monitoring the absolute signal strength significantly reduces the sensitivity range of the receiver compared to $C/N_{0}$ monitoring. On the other hand, authors in [47] propose spoofing detection based on the Improved Ratio combined with Carrier-to-Noise Moving Variance $\left(C/N_{0}-MV\right)$. This method gives the best results when the fake signal has 5 dB over the authentic signal with a detection rate of 98% whereas the classical SQM method gives only 30%.

**Figure 5:**
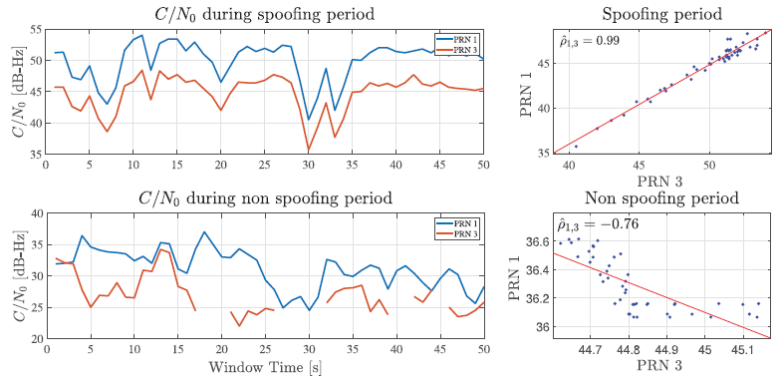
Comparison of $C/N_{0}$ for different satellites with and without spoofing attack [70].

In [39], authors use a combination of a few methods for spoofing detection. Spoofed GNSS signals were detected based on correlation peaks and phase differences between fake and authentic signals. Experimental results of smartphone susceptibility to a simplified spoofing attack are presented in [70]. Figure 5 shows the comparison of $C/N_{0}$ values for GPS PRN 1 and PRN 3 during (top) and without (bottom) a spoofing attack for Xiaomi Redmi 8. During a spoofing attack, the $C/N_{0}$ for both satellites is in the range of 35–55 dB-Hz while in conditions without an attack, a noticeable difference is visible in which $C/N_{0}$ has values of 20–40 dBHz with a slight trend of decrease and discontinuity at lower values. The correlation between values for both cases is confirmed by linear regression and Pearson’s correlation coefficient.

In the case of a spoofing attack, there is a higher correlation between the values with a coefficient of 0.99, while without an attack there is a low correlation with a coefficient of −0.76 due to data discontinuity and different trends. The sensitivity effects of smartphones are reflected through their raw measurements of, e.g., $C/N_{0}$, pseudorange, and position estimates. The impact of spoofing attacks on smartphones is analyzed in [71]. The authors suggest techniques to increase security such as using cheap acceleration sensors.

#### 3.1.3. Antenna Array Processing

In addition to spoofing detection with one antenna, an approach with the antenna array is also often used. Antenna array is often intertwined with spoofing detection with the help of DoA, which will be discussed later. Authors in the paper [25] present their antenna array-based method for spoofing detection. Their method for spoofing detection is based on the estimation of the DoAs of the received signal using compressed sensing methodology. The authors conduct their research on sophisticated spoofing attack scenarios and use a GNSS receiver with an array antenna and a spoofer with its own GNSS receiver. They suppose that the location of the target is known by the spoofer. DoAs of real and spoofed signals are estimated by the target GNSS receiver. The target GNSS receiver uses the spatial characteristics of the spoofing signal to estimate its direction of arrival. By using computer simulations, the authors confirm that their method can detect spoofing attacks successfully. In the paper [49], authors also focus on antenna array detection methods and use spatial components—their method for spoofing detection is based on a comparison of steering vectors related to received spatial components. The success of the proposed method is also confirmed with simulations. In the paper [51], the authors propose the use of three low-cost collinear antennas to detect GNSS spoofing. This approach is suggested for two reasons: traditional multi-antenna counterfeit detection methods are limited in application scenarios and using more antennas brings high costs. Simulation results for this method have high effectiveness. Unlike them, authors in [52] use a six-array spoofing-interference-monitoring array antenna. Their antenna successfully detects and identifies spoofing interference sources by monitoring the relevant peaks and combining an airspace-trapping algorithm. Authors in [53] use a multi-antenna GNSS receiver, and propose a model for spoofing detection in a way to compare and statistically test the measured DoAs in relation to the expected DoAs.

Authors in [54] use moving array antenna to localize GNSS spoofing sources. Firstly, the deceptive signals are separated from authentic signals based on a double-differenced carrier phase and then the original carrier phase single-difference data of the spoofing signal from multiple observation points is fused through a moving antenna array to directly localize the spoofing interference. This approach effectively avoids data correlation of the traditional two-step methods for DoA estimation parameters. Also, it provides the location accuracy of spoofing interference and the robustness of the method. Unlike the other approaches, authors in [50] propose a coprime array-based method for spoofing detection with a small time offset. This approach also estimates the DoA for the fake signal and indicates the spoofing source presence. Compared with other methods, this method achieves better performance in DoA estimation accuracy and does not perform the complex despreading and acquisition stage of the receiver since it is implemented on raw digital baseband signals.

### 3.2. Data Bit Methods

#### 3.2.1. Time of Arrival (ToA)

**Figure 6:**
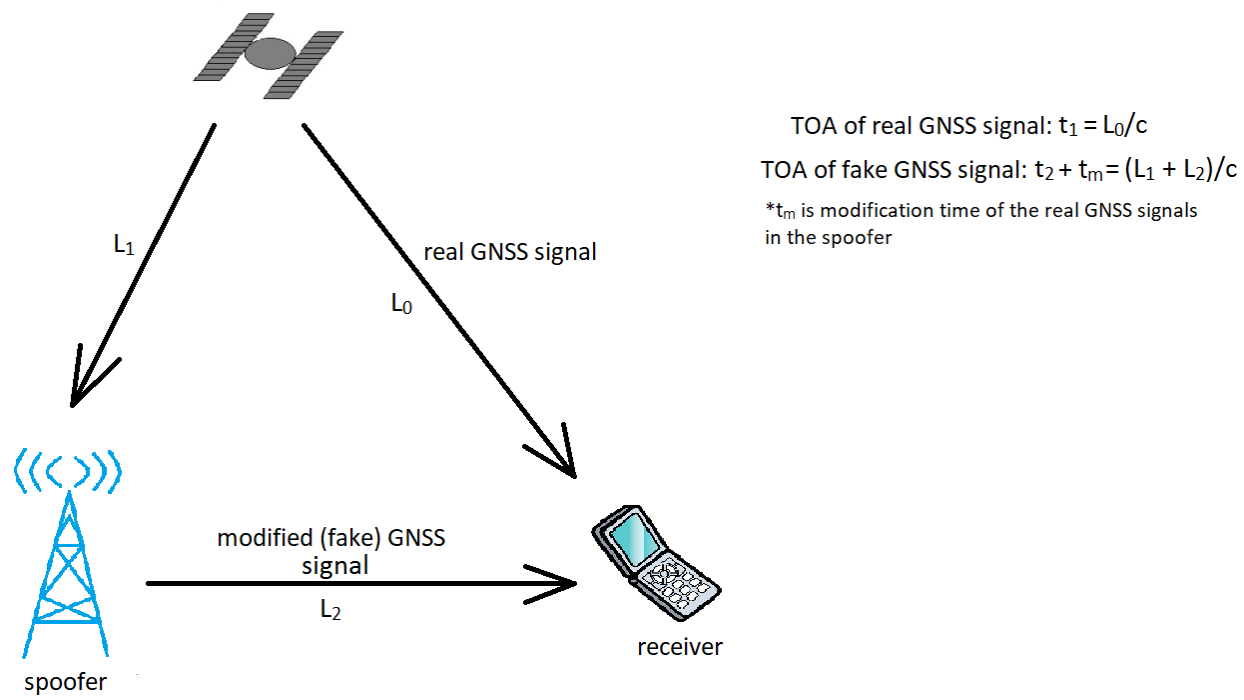
Time of arrival of real and fake GNSS signal (* denotes that tm is modification time of the real GNSS signals in the spoofer).

Authors in [20] say that GNSS positioning is based on ToA ranging. ToA is based on calculating the signal’s time propagation from the sender to the receiver. Spoofed signals have a longer time of arrival than real signals. Authors in the paper [55] propose a spoofing detection system based on time difference of arrival estimation (TDOAE). Their system consists of two receivers. Figure 6 shows the difference between the real and spoofed signal in accordance with the ToA.

#### 3.2.2. Direction of Arrival (DoA)

Spoofing detection is especially hard when a high-quality spoofer is used for the spoofing attack. It is not easy to discriminate between authentic and fake satellite signals in cases where all simulated signals have high fidelity. In cases like this, Controlled reception pattern antennas (CRPA) are used as the best option for defense—spoofer generates and transmits all simulated (spoofed) signals from the same location (one source), unlike authentic satellite signals which come from different sources (different satellites) from the sky. CRPA antenna rejects the signals if they come from the same direction because those kind of signals are probably fake ones.

Authors in [21] rely on the monitoring of two characteristics for the detection of spoofing:

1. The signal power from the spoofer compared to the power of the real satellites. This is discussed below in another section.
2. The DoA of the fake signals is different compared to the DoA of signals that come from the real satellites. Because of this claim, the authors in this paper compare the expected DoAs from different PRNs to detect if spoofing is present. Simulation results show that their presented algorithm detects spoofing well in spoofing scenarios with a single source of spoofing and even in scenarios with multiple sources of spoofing.

In the paper [56], the authors present a prototype of equipment for detecting spoofing attacks and determining the DoA and show that the presented prototype effectively detects spoofed signals in open environments even though the presented prototype is based on Low-Cost Commercial Board Components. The use of low-cost components is even mentioned as an advantage of the presented prototype. The implementation of a direction-finding curve is no longer limited to expensive antenna arrays. One of the disadvantages is that in low-elevation situations, the accuracy of the DoA calculation is relatively poor, which may be limited by the planar geometric configuration of the antennas in their article.

Since current detection methods based on DoA require multiple antennas/receivers, which leads to high costs and complexity, authors in [57] use a single rotating antenna for spoofing detection based on the intersection angle between two directions of arrival (IA-DoA). IA-DoA is estimated between a pair of signals by using the $C/N_{0}$ and Carrier Phase Single Difference (CPSD) of the received signal. This method proves effective for spoofing detection and performance detection improvement. Authors in [58] also use a single rotating antenna for signal spoofing detection. The proposed method is based on the improved probabilistic neural network (IPNN) which is used for classification. An accuracy of 98.84% is achieved.

In addition to all the mentioned ways of using DoA for detecting spoofing signals, the authors in [59] use DoA in combination with other methods to detect spoofing signals.

#### 3.2.3. NMEA Messages Analysis

The authors in [22] propose an approach based on the use of NMEA messages from GNSS receivers (smartphones and commercial ublox receivers) for the detection and identification of suspected potentially fake signals. NMEA 0183 messages contain information about visible satellites, the position of the receiver, speed and time, and their processing does not require significant processing. By using NMEA messages, the large computer loads required for obtaining and processing raw measurements are bypassed. Table 2 shows the types of NMEA messages and their descriptions. Three different scenarios were observed: in the first scenario the attacker emulated a drive that starts from a building and makes a loop around the nearby area, in the second scenario the attacker moves away from the building and returns to the start and the third scenario is the same as the second except that the attacker has additional damping. In the first scenario, the locations of all smartphones are successfully spoofed. Although the smartphones were in a stationary position on a table inside the building, the NMEA messages recorded that the devices were in motion in the surrounding area. For the second scenario, the spoofing attack affected the positioning accuracy but the fully expected fake trajectory was not observed, while for the third scenario, the spoofing attack was successful and the expected fake trajectory was observed. Although the devices are in a stationary state, logs noted that they are in a dynamic state under a spoofing attack. NMEA messages are also used for spoofing detection in maritime networks. In [60], authors present a novel low-cost framework MAritime Nmea-based Anomaly detection (MANA) for GPS spoofing detection also based on NMEA 0183 messages.

### 3.3. Positioning Methods Based on Pseudorange Measurements

Pseduorange is an approximation of the distance between a satellite and a GNSS receiver and is used to resolve positioning errors. GNSS receiver attempts to measure the ranges of (at least) four satellites as well as their positions when their positional data were transmitted. Pseudoranges are calculated by multiplying the speed of light with the time needed for each signal to reach the receiver.

Since spoofing can mislead the target receiver in reporting the wrong position and time, and fake signals that come from the different emitting sources are difficult to detect, authors in [61] propose spoofing detection by using pseudo-range double-differences (PRDD) measurements of two receivers. This approach detects spoofing signals by analyzing the differences between PRDD measurements and expected PRDD estimations with a detection probability of 99.99% and a false alarm rate of 0.001.

Most works focus on identifying the spoofing when it is under attack that is from the individual receiver side. A novel spoofing network monitoring (SNM) mechanism that detects the spoofing within an area and based on a different time difference of arrival (TDOA) between fake and authentic signals is proposed in [26]. TDOA is measured as the differential pseudorange to carrier frequency ratio. TDOAs of fake signals coming from the common spoofer are identical while those of authentic signals from different directions are dispersed.

Considering that array antenna methods use more than one antenna/receiver, in [27] authors present a method for detecting all kinds of spoofing attacks based on pseudorange differences for a single receiver. The authenticity of the signal is verified by comparing the results of the proposed method with the traditional least squares method. If spoofing is present, the comparison results of these two methods differ. Simulation results on the TEXBAT dataset confirm the feasibility and effectiveness of the proposed method.

Spoofing detection based on pseudorange measurements, used as features in machine learning methods is presented in the following subsection.

### 3.4. Radio Frequency Fingerprinting

Radio Frequency Fingerprinting in a GNSS system is a method used to identify and discriminate between authentic and spoofed signals by analyzing unique signal features to detect anomalies that indicate spoofing attacks. Lately, RFF methods become very popular for the purpose of identifying authentic transmitters and discriminating them from malicious transmitters, such as spoofers and jammers, especially in the context of non-GNSS transmitters (Wi-Fi, Internet of Things, etc.). In the context of GNSS transmitters, these methods are still in their infancy and have not been addressed so much to the best of the authors’ knowledge. Authors in [28] present an overview of RFF methods for spoofing detection in GNSS receivers. Also, they propose an approach for the RFF-based pre-correlation spoofing detection and transmitter identification. Their approach consists of four steps: relevant features identification, feature-extraction transform, data pre-processing, and classifier stage. The fourth step is based on machine and deep learning classification methods (SVM, KNN, CNN). Their analysis shows that a combination of different features in the SVM method gives the best results. In [29], authors also use machine learning methods—SVM and logistic regression for fingerprints (features) classification to identify if the recorded signal is authentic or spoofed. They achieve very high accuracy above 90% with the applied methods. SVM is also used in the RFF concept in [30] for pre- and post-correlation classifications of authentic and fake signals on three different datasets. Pre-correlation classification has higher accuracy (99.99%) than post-correlation classification (87.72%) due to the harder discrimination of RF fingerprints in the post-correlation domain (because of the additional filtering stages). Authors in [31] propose their framework based on a convolutional autoencoder for spoofing detection with high accuracy in the post-correlation domain. They verify their framework by performing three comparative experiments on the TEXBAT dataset. In [72], authors propose a GNSS spoofing detection method for RFF identification through simulations in ideal conditions. Compared to other methods, their method extracts the RFF from the received signals autonomously by exploiting deep learning, and manual feature selection is avoided with this approach. Two classification methods based on deep learning for RFF identification are evaluated. The first method uses only deep learning to learn the physical layer characteristics of the signal and the second one aims to extract RFF in the time-frequency domain. Their results show that the proposed method is efficient for the spoofing detection.

### 3.5. Machine and Deep Learning Methods

Machine learning can be combined with classical observation parameters and using a software-defined radio. For example, in the paper [70] the authors present experimental results of smartphone sensitivity to simplified spoofing attacks. The effects of smartphone sensitivity are manifested through raw measurements of parameters such as $C/N_{0}$, AGC, pseudorange, and position estimates. The authors reproduce two scenarios of a simplified spoofing attack. The spoofing attack lasts from 0 to 350 s. At the moment t = 350 s when the attack by faking it closes, the AGC value increases to its initial level as can be seen from the picture. The jump in AGC values for the Redmi 8 device may be due to the loss of hooking to authentic signals and re-tracking and hooking to fake signals. The great strength and durability of fake signals may be a factor in determining gaps in measurements. For example, if the fake signal is sufficiently strong and stable, the GNSS receiver may lose connection or “hang” on the signals for a long period, which results in a gap in GNSS measurements. On the other hand, if the spoofed signal is weak and less persistent, the receiver can keep latching onto the authentic signals and produce a continuous output, despite the presence of spoofed signals. Different receivers have different sensitivities and other features that affect resistance to spoofing attacks. A variety of datasets have been used in ML studies. These datasets can be publicly available (can be verified) or private (not shared with other researchers). The most popular publicly available datasets for spoofing scenarios and detection are TEXBAT and OAKBAT. Many papers use these datasets for the validation and verification of their methods. There are three categories of datasets [63]:

1. Real data includes raw data from smartphones, GNSS stations, and receivers.
2. Simulated data include SDR and software receiver, e.g., Spirent simulator [38].
3. Combination of real and simulated data is the most common case.

**Figure 7:**
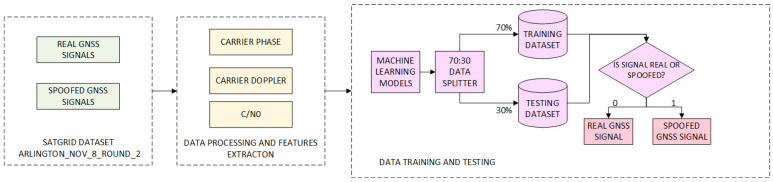
Block diagram for spoofing detection using ML methods [15].

Figure 7 shows the flow chart of the machine learning model for signal classification. The first step is to collect a dataset (authentic and fake signals). In the second step, the parameters that will be used to classify the signal are extracted. The last step is the application of machine learning methods, i.e., training and testing the model on the collected data. As a result, the model classifies the signals into authentic and fake based on the parameters used for training and testing. The described steps can be applied to all ML methods.

In [10], the authors compare the performance of several supervised models with the performances of unsupervised models in terms of accuracy, detection probability, fake detection probability, fake alarm probability, processing time, training time, prediction time, and memory size. The results show that classification and regression decision tree models outperform other supervised and unsupervised models in detecting and classifying GPS spoofing attacks. In [11,12], the authors compare the performance of several ML algorithms in detecting GPS signal spoofing attacks. The authors in [11] perform K-fold analysis to select the best ML algorithm among several ML algorithms. Based on their results, the SVM method with a polynomial kernel outperforms other methods. On the other hand, the results and analysis of ML algorithms in [12] show that algorithms based on decision trees give better results compared to SVM (linear and radial), KNN, and other analyzed algorithms.

In [62], the authors propose the detection of fake GNSS signals using the SVM machine learning method with the combination of real and simulated datasets to verify and validate the machine learning algorithms. The results show that the SVM method is a promising approach for fake signal detection. However, this research does not analyze the reasons for choosing certain parameters and the combination and preference for certain features. Most existing spoofing detection algorithms use the existing TEXBAT dataset published by the University of Texas [73], with relatively fixed scenarios. Albright et al. from Oak National Laboratory Ridge, USA, published another ready-made OAKBAT dataset [74] containing fake signals GPS and Galileo, providing multiple test scenarios to investigate the detection of spoofing attacks.

**Figure 8:**
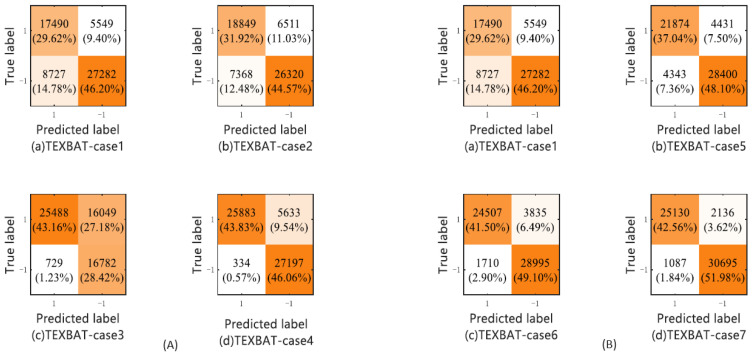
(**A**,**B**) Confusion matrix for spoofing attack detection in TEXBAT dataset [14].

The authors in [14] propose a GNSS multi-parameter joint detection method that is also based on the SVM method by processing and comparing the TEXBAT and OAKBAT datasets. The obtained results show a significant improvement in the performance of fake signal detection compared to traditional one-parameter methods. Authors in [15] compare the results of classification for 2 datasets—their own and publicly available SatGrid dataset [75]. On the other hand, the authors in Part I [16] use three synthetically generated (simulated) fake signal datasets with the Spirent simulator for training and verification and two datasets for model validation created using software-defined radios LimeSDR and HackRF. The authors use the C-SVM method of supervised machine learning to detect fake signals. In Part II [13], the authors supplement the experiments and results obtained in Part I. In addition to the laboratory-generated fake signal datasets used in Part I to train the model, real-time fake signal datasets were added in the training phase of the C-SVM method. Figure 8 shows the confusion matrix for the spoofing attacks detection using different parameters—(a) and a combination of different parameters—(b). It is evident from the pictures that the accuracy of SVM methods improved in case seven, in which all nine parameters were used, with 75.82% to 95.54%. SVM is proven as the most accurate method for the classification of signals in SatGrid dataset [15] with an accuracy of 99.7%. Next to SVM, KNN has an accuracy of 99.67%. In [66], authors also use a couple of supervised ML methods to detect GNSS fake signals on different scenarios of used datasets. Several of the ML methods have a classification f1-score exceeding 99% and the best results are achieved with Linear Regression and KNN for scenario 1 in which training and testing data are from the same dataset, and scenario 2 when training data is from the simulated dataset and testing data is from the recorded different dataset.

In a review paper [63], recommendations for researchers were given and it is concluded that ML methods are a promising approach for application in GNSS systems. Since unmanned aerial vehicles (UAV) are very sensitive to this type of attack, the authors in [64] give a comparison of several tree-based supervised machine learning models to detect spoofing attacks and collect real GPS signals using SDR. In [65], the authors evaluate five instance-based machine learning models for detecting fake GPS signals. Also, the authors use an SDR unit to collect and extract features of satellite signals and simulate three types of spoofing attacks (simplistic attack, intermediate attack, and sophisticated attack). The results show that Nu-SVM has the best performance. The authors in [48] propose navigation in the environment where a GNSS spoofing attack occurs of the signal taking into account the received power, correlation distortion function, and pseudoranges. Both real and fake measurements are used in the dataset. Machine learning displays authentic measurements from the available set using parameters such as received power and correlation function distortion. Several machine learning methods were used in the paper for the classification and detection of fake signals. Neural networks and linear SVM were shown to be the best methods with an accuracy of 98.20%.

To detect false signals at different power levels, authors in the paper [17] use Convolutional Neural Networks (CNNs). In their research, they generate the signal with the help of an advanced simulator and use different devices to interfere with the signal. They compare the effect of 5 pre-trained CNNs—AlexNet, GoogleNet, VGG-16, ResNet-18, and MobileNet-V2. The MobileNet-V2 method stands out compared to other techniques with an accuracy of 99.80%.

Authors in [18] also use deep neural networks for spoofing attack detection. They use Cross Ambiguity Function (CAF) images for signal spoofing detection. CAF gets computed by GNSS receivers. The authors implement a data-driven classifier through an image segmentation process. In addition, they consider a Gaussian mixture model approach to determine the number of false signals. The method they propose requires the use of multiple neural network models, which makes it computationally more demanding. Finally, the obtained results show that the proposed method has a very high success rate in detecting spoofing signals compared to previously known methods, and it stands out especially when it comes to moderate to high signal-to-noise ratios. In paper [19], authors use the neural network multi-layer perceptron neural network on selected metrics and combined datasets to classify artificially created spoofing scenarios.

## 4. Jamming and Spoofing Combination Detection Methods

**Figure 9:**
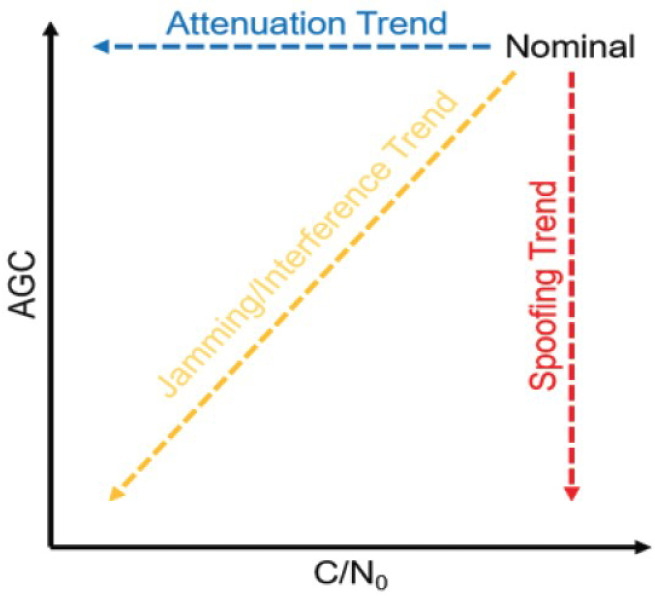
Expected trend for AGC and $C/N_{0}$ [77].

Since jamming and spoofing represent the main threats and security risks in the GNSS community, detection methods for these threats are similar and based on the same parameters. In the paper [76], the authors describe how the aforementioned parameters can be used to distinguish between fake and interfering signals. If both AGC and $C/N_{0}$ decrease, a jammed signal is more likely, and if AGC decreases and $C/N_{0}$ remains constant, a spoofed signal is more likely. If the AGC is constant, then any form of interference is unlikely, and a weak signal can be attributed to attenuation. In the paper [77], the authors propose a solution for the detection of jamming and spoofing attacks using the original parameters (among others AGC and $C/N_{0}$) of the location within Android. This solution increases the robustness of position and time calculations in Android systems and is implemented in the GNSSAlarm Android application that contains indicators for AGC and $C/N_{0}$. If the AGC drops below the set threshold and the $C/N_{0}$ drops to an equal amount or more, interference is likely and the corresponding indicators turn yellow as shown in the figure. If the same scenario occurs and the $C/N_{0}$ does not drop proportionally, the indicators turn red and warn of a spoofing attack as shown in Figure 9.

In [78], the authors propose a method for the detection of spoofing and signal jamming attacks based on automatic gain control and $C/N_{0}$ observations. A spoofing attack is likely to be detected when the AGC value decreases, and the $C/N_{0}$ is relatively constant or even increased. However, AGC is not sufficient to detect the presence of a fake signal, but only to raise a warning. Therefore, AGC should be used in combination with $C/N_{0}$.

Authors in [79] present their two-stage GNSS interference suppression scheme based on antenna arrays and show that it can successfully detect jamming and spoofing signals. Antenna array detection methods are one of the methods used for both jamming and spoofing detection. In the first phase, the authors focus on jamming removal by adopting subspatial projection for high-power jamming signals removal. In the second phase, the authors focus on low-power spoofing signals. In these low-power signals, the cyclo stationarity of the navigation signals was fully investigated to detect false signals and estimate the spatial power spectrum before the downscaling process. Subspatial projection mitigates spoofing signals and beamforming for all satellites and thus ensured that the strength of authentic signals was not overpowered by spoofing signals. This scheme even maximizes the power of the authentic signals. To prevent the loss of authentic signals, the authors in [80] present a method to find differences in background noise and delay between authentic and spoofed signals.

Table 3 shows an overview of the papers that use machine and deep learning methods discussed in our work. The table contains features and parameters used in the papers, the accuracy of applied methods, and the type of interference detected.

Like spoofing, the simplest method for jamming detection is based on observing power measurements usually through AGC and $C/N_{0}$ parameters [89]. $C/N_{0}$ measurements and sometimes AGC are provided by all grades of GNSS receivers, from low-cost to professional. The crowdsourcing method for jamming detection is proposed in [90]. Installing a GPS jam-to-noise detector in cellphones is suggested to detect jamming in time. Axell et al. in [91] also use smartphones as receivers for detecting jamming. They propose $C/N_{0}$-based Android application detectors and conclude that these detectors can work well in static scenarios since smartphones rely only on $C/N_{0}$ as an indicator. Proposed detectors are suitable in dynamic scenarios since they cannot distinguish between decreased GPS signal strength and increased interference. Another mobile phone crowdsourcing approach for jamming detection also called the J911 system, is presented in [92]. This system crowdsources the measurements from different smartphones and discriminates the natural signal degradation from intentional jamming based on AGC and $C/N_{0}$. The value of nominal AGC voltage needs to be known in order to be used (each device has its own nominal value). This system is tested on GPS and GLONASS signals. When the jammer turns on and gets closer to the smartphone, $C/N_{0}$ for both GPS and GLONASS decreases in a similar pattern. When the jammer moves away from the smartphone, $C/N_{0}$ returns to nominal values. $C/N_{0}$ measurements are validated by comparison to AGC measurements. Field trials show the usefulness of the specified approach. Since the jammer localization algorithm is not explicitly proposed in [92], Olsson et al. in [93] propose a novel jamming localization algorithm that is based on participatory sensing. The proposed algorithm automatically estimates all parameters and does not need any prior knowledge of the jammer or path loss model, and uses AGC and $C/N_{0}$ estimates or their combination from commercial receivers. Authors in [94] extend the algorithm from [93] for jammer localization by including only the received data during times when jamming is detected. On the other hand, in [93] all data is used and it might also include clean data that may affect the estimations in a bad way.

Previously mentioned papers refer to the static scenarios. In [95], authors present a novel Bayesian probabilistic method for jamming detection and localization on cellphones in static and dynamic scenarios. This approach can cope with multiple jammers and does not need prior information about the jammer’s power. The authors also introduce the GNSS coverage map that can help detect potential jammers within a city.

**Figure 10:**
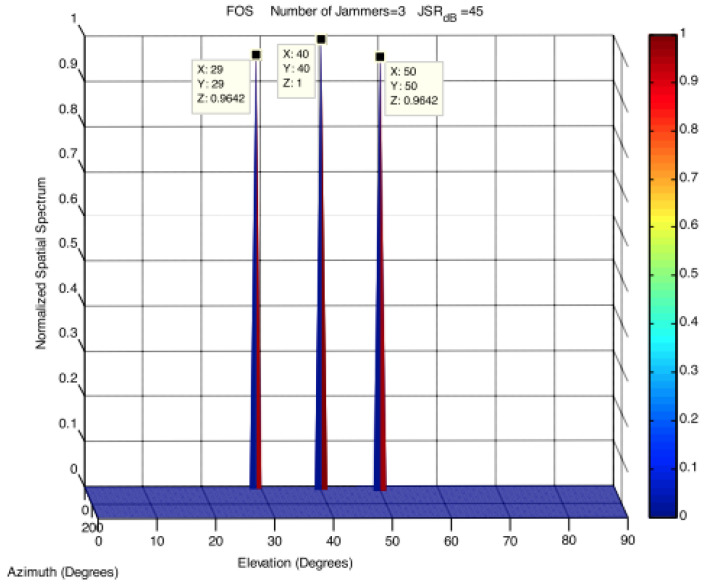
FOS DOA estimation for three jammers [96].

Osman et al. in [96] propose another approach for jamming detection based on DOA elevation and azimuth angle estimation for GPS jamming signals in challenging environments. DOA estimation is based on the fast orthogonal search (FOS) method. Their approach has significant improvement in the accuracy of detecting the number of jammers and their DOAs. Figure 10 shows the successful detection of three jammers arriving at jamming to signal ratio (JSR) of 45 dB. Power-based and direction-finding detectors for in-lab validation are considered in [97]. Considered detectors have good detection probabilities for JSR above −10 dB. Just as the widespread availability and affordability of SDRs has significantly advanced the development and deployment of GNSS spoofing detection systems, it has also advanced the development and deployment of GNSS jamming detection systems, as authors in [98] show. They also propose the use of signal powers from software-defined radio for real-time jamming detection.

Jamming threats are not rare even in maritime traffic. Authors in [99] give an overview of jamming threats and countermeasures techniques in order to improve navigation and positioning in maritime traffic. The authors emphasize the most advanced methods of signal processing, antenna array-based methods, and sensor fusion as the most effective methods for the mentioned problem. The German authorities have given permission to conduct an experiment on the Baltic Sea in which a jamming attack is carried out in maritime traffic. This experiment looks like one vessel is anchored and acts like the attacker, and the victim’s vessel is performing maneuvers in the vicinity of the attacker. The results of the conducted experiment showed that the area of influence of interference exceeds a radius of three kilometers, although its effect is not uniform.

As well as for spoofing, machine learning methods are also used for jamming signal detection. In the paper [81], the authors propose a classification method using SVM and CNN on the dataset composed of 61,800 different images and different jammer types. They suggest classifying the received signal into 6 classes. Using the SVM classifies the received signals into six classes and it does this with a very high accuracy of 94.90%. The use of neural networks gives a slightly lower classification accuracy of 91.36%. High classification accuracy is achieved on small dataset of images and not very complex parameters/network layer architectures. CNN is also used in [85] for single jamming classification with a classification accuracy of almost 100%. Other papers use multi-layer NN [82] with 99.3247%, SVM [100], CNN in [88], LSTM and CNN with the accuracy of 100% for both models without fading, with fading LSTM has a lower accuracy [83].

TWSVM algorithm is proposed in [84] for real-time jamming monitoring. Results indicate that TWSVM is faster than SVM in training (millisecond level) and classification speed (microsecond level).

MLP with particle swarm optimization is used in [101] for spoofing detection with the accuracy improvement of 2% and 4% compared to multihypothesis Bayesian classifier and Bayes-optimal rule classification. Features used for Signal power and correlation distortion function are features used for classifying signals into categories: multipath, jammed, spoofed, or interference-free signals.

Unmanned Aerial Vehicles are also compromised in terms of navigation and positioning. So, authors in [86] also use machine learning classification for signal jamming detection. Seven multi-class machine learning models with multiple outputs are trained and their training and testing are carried out. MLP model has optimal performance with a detection rate of 98.9%.

In the paper [87], it is also shown that machine learning methods give great results for signal jamming detection. In this paper, the use of federated learning (FL) is investigated. Authors use federated learning to train jamming signal classifiers locally on each device and thereby achieve privacy-preserving data training while collecting a huge amount of user’s data. The authors present results for the image classification of the spectrogram of the simulated GNSS signal under the threat of six different types of jammers. DME jammer provides the best accuracy of 99%.

## 5. Discussion and Conclusions

In this review, we deal with the topic of GNSS interferences and present methods for jamming and spoofing interference detection. In addition to an overview of spoofing detection methods, which some authors have already covered, e.g., [8], in this paper we also cover methods for jamming detection and jamming and spoofing combination detection methods in the last few years. The discussed papers highlight machine and deep learning methods, signal strength, and $C/N_{0}$ monitoring, and antenna array-based methods as reliable and widely used methods for jamming and spoofing detection. Machine learning methods enable users to detect and classify the signal in order to prevent interferences, especially in urban areas where such attacks are more common.

In this paper, the methods for detecting spoofing are discussed in detail and divided into several categories: signal processing methods, data bit methods, positioning methods, and machine and deep learning methods. Each category contains different types of methods. Besides machine and deep learning methods, signal strength and $C/N_{0}$ monitoring, and antenna array-based methods which we highlight as the most frequently used, there are more methods that we present and describe in this paper. Spoofing detection by ToA, DoA, and NMEA message analysis are data bit methods that are also often used. Except for spoofing, DoA usage for signal jamming detection is widespread. Detection with correlation peak monitoring and pseudoranges measurements are also described in this paper. Considering that Radio Frequency Fingerprinting methods have not been investigated and used much in the context of GNSS, application and further development of these methods have a great potential for contribution to the GNSS community.

Since GNSS systems are very complex and may have multiple potential sources of different interferences like jamming, spoofing, multipath, and unintentional interference from electronic devices, the simultaneous occurrence of all interferences or combination of multiple interferences makes it difficult to monitor and detect them in real-time. As jamming and spoofing technology develops rapidly and becomes a security risk in all aspects of life, especially in navigation and positioning applications, aircraft, and maritime, its detection becomes more challenging for the GNSS community. All these applications require high levels of reliability and accuracy to provide accurate positioning, navigation, and timing information at each moment. Another reason for focusing on detecting the combination of different interferences is improving signal processing techniques that can improve the robustness of GNSS receivers and make them more resistant to different interferences. The most important reason is related to security especially in the military when proactive and fast responses to spoofing and jamming attacks are required. Real-time detection enables fast responses to these threats and needed countermeasures can be deployed immediately. One of the future directions and challenges for the GNSS research community should be research and investigation for detecting combinations of different interferences in real time to ensure the reliability, accuracy, and robustness of GNSS systems for the reasons stated above. Different detection and localization methods may be combined to improve the detection, classification, and localization accuracy with an emphasis on machine and deep learning methods which are currently trending and promising in their use in GNSS. On the other hand, ML and DL methods still have a limited use in the industry.

One of the limitations for researchers is the lack of publicly available datasets. To the authors’ best knowledge, two relevant and high-quality datasets are mostly used for validation and verification, TEXBAT and OAKBAT. These datasets represent different spoofing scenarios. However, the GNSS community will benefit from datasets that will include the combinations of different interferences and this may be a potential direction of research.

Some detection methods use a large amount of equipment which results in high costs, but advances are seen in the form of the use of less interference detection equipment and the use of low-cost components. It is believed that in the future hardware costs will be minimized as much as possible. In addition, there are not many approaches that rely on a single rotating antenna so using the stated approach instead of array antenna technology would reduce the costs.

Table 4 compares jamming and spoofing according to some criteria such as the impact on the system, cost, required equipment, mechanism of attack, and detection complexity. It can be concluded that jamming is less complex, easier to detect, and requires simple equipment.

Table 5 presents a comprehensive overview of nine different jamming and spoofing detection methods, analyzing their working principles, spoofing/jamming detection ability, advantages and disadvantages. Each method has specific characteristics that make it suitable for certain scenarios and needs. In conclusion, the spoofing/jamming method choice should be based on specific system requirements, including the required level of precision, the ability to detect different threats, the available resources and the environment in which the system operates. Combining multiple methods often provides the best protection, allowing for a balance between the advantages and disadvantages of individual techniques. Another interesting approach that may be used for interference detection is different statistical methods that can monitor the distortions in correlation function and detect different fluctuations in the power of signal caused by different interferences. This approach has low computational complexity because there is no need for additional changes in the receiver’s configuration, which has a great potential application in receivers equipped with blocks for detecting interferences.

## Figures and Tables

**Table 1:** Categories of Spoofing Detection Methods [24].

Method	Type	Description	Reference
Signal Processing Methods		Signal Quality Monitoring (SQM),	
	Correlation Peak Monitoring	Monitoring the distribution of correlation peak and function	[23,39,40,41,42,43,44]
	Power-based Methods	Signal power, Automatic Gain Control (AGC) and $C/N_{0}$ monitoring	[24,45,46,47,48]
	Antenna Array Processing	Spoofing detection by using antenna array	[25,49,50,51,52,53,54]
Data Bit Methods	ToA	Calculating the signal’s time propagation from the sender to the receiver	[20,55]
	DoA	Monitoring the direction from which the signals arrive at the receiver	[21,56,57,58,59]
	NMEA Messages Analysis	consistency check among satellite navigation messages	[22,60]
Positioning Methods	Pseudorange Measurements	integrity check among different pseudorange measurements	[26,27,61]
Machine and Deep Learning Methods	Different machine and deep learning methods	Model training and testing by using different parameters and a different machine and deep learning methods	[10,11,12,13,14,15,17,18,48,62,63,64,65,66]

**Table 2:** Definition of NMEA messages provided by GNSS receivers [22].

NMEA Message Type	Description
GSV	GNSS satellites in view—PRN, Elevation, Azimuth, $C/N_{0}$
GSA	GNSS Dilution of Precision (DOP) and active satellites
GGA	GNSS fix data—Time, Position, DOP
RMC	Recommended minimum specific data—Time, Position, Velocity
VTG	Track made good and ground speed—Velocity, Heading
GRS (not available for smartphones)	Range residuals for active satellites

**Table 3:** ML and DL methods with used parameters and accuracy in the state of art.

ML/DL Method	Features/Parameters	Accuracy in %	Reference	Interference
SVM, NN	average power, correlation distortion	98.20	[48]	spoofing
KNN	phase difference	95 for delay greater than 0.4 chip	[23]	spoofing
GAN	phase difference, correlation peaks	98	[39]	spoofing
Kernel Naive Bayes, Boosted Trees	average power, correlation distortion	80.75	[44]	jamming & spoofing
Decision Trees	$C/N_{0}$	greater than 98.48, for $C/N_{0}$ between 40 dBHz and 50 dBHz	[12]	spoofing
SVM (radial basis function)	$C/N_{0}$	97.8	[62]	spoofing
SVM	correlation function, $C/N_{0}$, pseudorange Doppler consistency, PVT solving residuals, clock difference, clock drift	97.24	[14]	spoofing
C-SVM	lock time, pseudoranges, $C/N_{0}$, carrier Doppler frequency	98.77	[16]	spoofing
XGBoost	$C/N_{0}$, pseudoranges, carrier phase, receiver time, prompt, early and late correlator	95.52	[64]	spoofing
Nu-SVM	$C/N_{0}$, pseudoranges, carrier phase, carrier Doppler, receiver time, early and late correlator	92.78	[65]	spoofing
MobileNet-V2 CNN	power level on scalogram images	99.80	[17]	jamming
Deep neural network	correlation function, $C/N_{0}$	not applicable	[18]	spoofing
Multi-Layer Perceptron (MLP) neural network	Q-channel SQM metric, $C/N_{0}$, early–late phase metric	82	[19]	spoofing
SVM	$C/N_{0}$, carrier phase, carrier Doppler	99.7	[15]	spoofing
SVM, CNN	70% of the strongest features used	94.90 and 91.36	[81]	jamming
multi-layer NN	phase, energy and correlation distribution function	99.3247	[82]	spoofing
Long Short-Term Memory (LSTM), CNN	signal to noise ratio, correlation function	100	[83]	spoofing
Twin SVM algorithm (TWSVM)	$C/N_{0}$, correlator output, correlator output power	100	[84]	different types of interferences
CNN	13 different structure parameters	100	[85]	jamming
MLP	14 different features	98.9	[86]	jamming
Distance Measuring Equipment (DME)	6 different features	over 99	[87]	jamming
CNN with SVM and LR	ImageNet dataset	98	[88]	jamming

**Table 4:** Jamming and spoofing comparison.

Criteria	Jamming	Spoofing
Definition	Emission of interfering signals	Creation of fake signals
Impact on System	Complete/partial communication loss	Incorrect data, unnoticed for longer periods
Cost	Lower cost	Higher cost
Resources Required	Simple equipment	Sophisticated equipment
Mechanism of Attack	Overpowers with noise or signals	Generates fake but convincing signals
Detection Complexity	Easier to detect due to signal loss, less complex	Harder to detect, signal strength remains, more complex

**Table 5:** Comparison between jamming and spoofing detection methods.

Method	Principle	Spoofing Detection	Jamming Detection	Advantage	Disadvantage
Signal Power Monitoring	monitoring sudden changes in signal’s power	Yes	Yes	Easy implementation	hard to discriminate between jamming and natural signal variations
Carrier-to-Noise Ratio	observing $C/N_{0}$ degradations	Yes	Yes	high values reveal the presence of spoofed signals	may not detect sophisticated spoofing attacks
Automatic Gain Control	monitoring AGC levels	No	Yes	very simple, integration into the existing receivers	can detect spoofing when used in combination with another parameter
Correlation Peak Monitoring	monitoring correlation peaks of GNSS signals	Yes	No	detecting spoofing with abnormal correlation peaks	hard to detect sophisticated spoofing
Direction of Arrival Monitoring	comparison of signals’ angles of arrivals	Yes	No	able to detect fake signals from different directions	complex implementation and high cost since multiple antennas are required
Time of Arrival Monitoring	monitoring the expected time of arrival of signals	Yes	No	high accuracy in detecting spoofed signals	requires precise time synchronization and reference signals
Multi-Receiver Techniques	comparing data from multiple GNSS receivers	Yes	No	high accuracy	high cost and complexity since multiple receivers are required
Code and Carrier Phase Monitoring	monitoring the alignment and consistency between the code phase and carrier phase of the signal	Yes	Yes	high accuracy in detecting both spoofing and jamming	requires complex algorithms and high computational resources
Machine Learning	using different machine learning for detecting interferences	Yes	Yes	high detection accuracy	computationally complex

## Data Availability

No new data were created or analyzed in this study. Data sharing is not applicable to this review.

## Abbreviations

The following abbreviations are used in this manuscript:

AGC	Automatic Gain Control
BPSK	Binary Phase Shift Keying
CAF	Cross Ambiguity Function
$C/N_{0}$	Carrier-to-Noise Ratio
$C/N_{0}-MV$	Carrier-to-Noise Moving Variance
CNN	Convolutional Neural Networks
CPSD	Carrier Phase Single Difference
CRPA	Controlled Reception Pattern Antenna
DoA	Direction of Arrival
DL	Deep Learning
DME	Distance Measuring Equipment
DOP	Dilution of Precision
ETSI	European Telecommunications Standards Institute
FL	Federated Learning
FOS	Fast Orthogonal Search
GAN	Generative Adversarial Network
GNSS	Global Navigation Satellite System
GPS	Global Positioning System
IA-DoA	Intersection Angle between Two Directions of Arrival
IPNN	Improved Probabilistic Neural Network
ISO	International Organization for Standardization
JSR	Jamming-to-Signal Ratio
KNN	K-Nearest Neighbors
KS	Kolmogorov–Smirnov test
LSTM	Long Short-Term Memory
ML	Machine Learning
MLP	Multi-Layer Perceptron
NMEA	National Marine Electronics Association
OAKBAT	Oak Ridge Spoofing and Interference Test Battery
PRDD	Pseudo-Range Double-Differences
PRN	Pseudo-Random Noise Code
PVT	Position, Velocity, and Time
RFF	Radio Frequency Fingerprinting
SCPC	Spoofing Correlation Peak Cancellation
SDR	Software Defined Radio
SNM	Spoofing Network Monitoring
SQM	Signal Quality Monitoring
SVM	Support Vector Machine
TDOA	Time Difference of Arrival
TDOAE	Time Difference of Arrival Estimation
ToA	Time of Arrival
TEXBAT	Texas Spoofing Test Battery
TWSVM	Twin SVM Algorithm
UAV	Unmanned Aerial Vehicles
USRP	Universal Software Radio Peripheral

## References

[1] Novatel What Are Global Navigation Satellite Systems?. https://novatel.com/tech-talk/an-introduction-to-gnss/what-are-global-navigation-satellite-systems-gnss.

[2] Space Systems—Requirements for Global Navigation Satellite System (GNSS) Positioning Augmentation Centers.

[3] ETSI TS 103 246-5 V1.1.1 (2016-01) and V1.3.1 (2020-10). Satellite Earth Stations and Systems (SES). GNSS-Based Location Systems, Part 5: Performance Test Specification. https://portal.etsi.org/Services/editHelp/Search/FAQs/TEDDI.

[4] Psiaki M.L., Humphreys T.E., Stauffer B. (2016). Attackers can spoof navigation signals without our knowledge. Here is how to fight back GPS lies. IEEE Spectr..

[5] Wu Z., Zhang Y., Yang Y., Liang C., Liu R. (2020). Spoofing and Anti-Spoofing Technologies of Global Navigation Satellite System: A Survey. IEEE Access.

[6] Humphreys T.E., Ledvina B.M., Psiaki M.L., O’Hanlon B.W., Kintner P.M. Assessing the Spoofing Threat: Development of a Portable GPS Civilian Spoofer. Proceedings of the 21st International Technical Meeting of the Satellite Division of the Institute of Navigation (ION GNSS Conference).

[7] Humphreys T.E. (2013). Detection Strategy for Cryptographic GNSS Anti-Spoofing. IEEE Trans. Aerosp. Electron. Syst..

[8] Meng L., Yang L., Yang W., Zhang L. (2022). A Survey of GNSS Spoofing and Anti-Spoofing Technology. Remote Sens..

[9] Turner M., Wimbush S., Enneking C., Konovaltsev A. Spoofing Detection by Distortion of the Correlation Function. Proceedings of the 2020 IEEE/ION Position, Location and Navigation Symposium (PLANS).

[10] Khoei T.T., Gasimova A., Ahajjam M.A., Shamaileh K.A., Devabhaktuni V., Kaabouch N. A Comparative Analysis of Supervised and Unsupervised Models for Detecting GPS Spoofing Attack on UAVs. Proceedings of the 2022 IEEE International Conference on Electro Information Technology (eIT).

[11] Shafique A., Mehmood A., Elhadef M. (2021). Detecting Signal Spoofing Attack in UAVs Using Machine Learning Models. IEEE Access.

[12] Gallardo F., Yuste A.P. (2020). SCER Spoofing Attacks on the Galileo Open Service and Machine Learning Techniques for End-User Protection. IEEE Access.

[13] Semanjski S., Semanjski I., De Wilde W., Gautama S. (2020). Use of Supervised Machine Learning for GNSS Signal Spoofing Detection with Validation on Real-World Meaconing and Spoofing Data—Part II. Sensors.

[14] Chen Z., Li J., Li J., Zhu X., Li C. (2022). GNSS Multiparameter Spoofing Detection Method Based on Support Vector Machine. IEEE Sens. J..

[15] Radoš K., Brkić M., Begušić D. GNSS Signal Classification based on Machine Learning Methods. Proceedings of the 2024 47th MIPRO ICT and Electronics Convention (MIPRO).

[16] Semanjski S., Semanjski I., De Wilde W., Muls A. (2020). Use of Supervised Machine Learning for GNSS Signal Spoofing Detection with Validation on Real-World Meaconing and Spoofing Data—Part I. Sensors.

[17] Elango A., Ujan S., Ruotsalainen L. Disruptive GNSS Signal detection and classification at different Power levels Using Advanced Deep-Learning Approach. Proceedings of the 2022 International Conference on Localization and GNSS (ICL-GNSS).

[18] Borhani-Darian P., Li H., Wu P., Closas P. (2024). Detecting GNSS spoofing using deep learning. EURASIP J. Adv. Signal Process..

[19] Marchand M., Toumi A., Seco-Granados G., López-Salcedo J.A. Machine Learning Assessment of Anti-Spoofing Techniques for GNSS Receivers. Proceedings of the WIPHAL 2023: Work-in-Progress in Hardware and Software for Location Computation, CEUR Workshop Proceedings.

[20] Truong V., Vervisch-Picois A., Rubio Hernan J., Samama N. (2023). Characterization of the Ability of Low-Cost GNSS Receiver to Detect Spoofing Using Clock Bias. Sensors.

[21] Yang Q., Chen Y. A GPS Spoofing Detection Method Based on Compressed Sensing. Proceedings of the 2022 IEEE International Conference on Signal Processing, Communications and Computing (ICSPCC).

[22] Lee D.-K., Miralles D., Akos D., Konovaltsev A., Kurz L., Lo S., Nedelkov F. Detection of GNSS Spoofing using NMEA Messages. Proceedings of the European Navigation Conference (ENC).

[23] Li J., Li W., He S., Dai Z., Fu Q. Research on Detection of Spoofing Signal with Small Delay Based on KNN. Proceedings of the 2020 IEEE 3rd International Conference on Electronics Technology (ICET).

[24] Jafarnia-Jahromi A., Broumandan A., Nielsen J., Lachapelle G. (2012). GPS vulnerability to spoofing threats and a review of antispoofing techniques. Int. J. Navig. Observ..

[25] Lee Y.-S., Yeom J.S., Jung B.C. A Novel Array Antenna-Based GNSS Spoofing Detection and Mitigation Technique. Proceedings of the 2023 IEEE 20th Consumer Communications & Networking Conference (CCNC).

[26] Zhang Z., Zhan X. (2016). GNSS Spoofing Network Monitoring Based on Differential Pseudorange. Sensors.

[27] Liu K., Wu W., Wu Z., He L., Tang K. (2018). Spoofing Detection Algorithm Based on Pseudorange Differences. Sensors.

[28] Wang W., Aguilar Sanchez I., Caparra G., McKeown A., Whitworth T., Lohan E.S. (2021). A Survey of Spoofer Detection Techniques via Radio Frequency Fingerprinting with Focus on the GNSS Pre-Correlation Sampled Data. Sensors.

[29] Morales-Ferre R., Wang W., Sanz-Abia A., Lohan E.-S. (2020). Identifying GNSS Signals Based on Their Radio Frequency (RF) Features—A Dataset with GNSS Raw Signals Based on Roof Antennas and Spectracom Generator. Data.

[30] Wang W., Lohan E.S., Sanchez I.A., Caparra G. Pre-correlation and post-correlation RF fingerprinting methods for GNSS spoofer identification with real-field measurement data. Proceedings of the 10th Workshop on Satellite Navigation Technology (NAVITEC).

[31] Zhang X., Huang Y., Tian Y., Lin M., An J. (2023). Noise-Like Features-Assisted GNSS Spoofing Detection Based on Convolutional Autoencoder. IEEE Sens. J..

[32] Mukherji V., Chandele A.K.S. GNSS Jamming: An Omnipresent Threat. Geospatial World. https://www.geospatialworld.net/prime/special-features/gnss-jamming-an-omnipresent-threat/.

[33] Li X., Chen L., Lu Z., Wang F., Liu W., Xiao W., Liu P. (2023). Overview of Jamming Technology for Satellite Navigation. Machines.

[34] Songala K.K., Ammana S.R., Ramachandruni H.C., Achanta D.S. Simplistic Spoofing of GPS Enabled Smartphone. Proceedings of the 2020 IEEE International Women in Engineering (WIE) Conference on Electrical and Computer Engineering (WIECON-ECE).

[35] Radoš K., Brkić M., Begušić D. Vulnerability of Smartphones on GNSS Simplistic Spoofing Attack. Proceedings of the 2024 47th MIPRO ICT and Electronics Convention (MIPRO).

[36] Psiaki M.L., Humphreys T.E. (2016). GNSS Spoofing and Detection. Proc. IEEE.

[37] Garbin Manfredini E. (2017). Signal Processing Techniques for GNSS Anti-Spoofing Algorithms. Ph.D. Thesis.

[38] Broumandan A., Kennedy S., Schleppe J. Demonstration of a Multi-Layer Spoofing Detection Implemented in a High Precision GNSS Receiver. Proceedings of the 2020 IEEE/ION Position, Location and Navigation Symposium (PLANS).

[39] Li J., Zhu X., Ouyang M., Li W., Chen Z., Fu Q. (2021). GNSS Spoofing Jamming Detection Based on Generative Adversarial Network. IEEE Sens. J..

[40] Yang B., Tian M., Ji Y., Cheng J., Xie Z., Shao S. (2022). Research on GNSS Spoofing Mitigation Technology Based on Spoofing Correlation Peak Cancellation. IEEE Commun. Lett..

[41] Fang J., Yue J., Xu B., Hsu L.-T. (2023). A post-correlation graphical way for continuous GNSS spoofing detection. Measurement.

[42] Zhou W., Lv Z., Li G., Jiao B., Wu W. (2024). Detection of Spoofing Attacks on Global Navigation Satellite Systems Using Kolmogorov–Smirnov Test-Based Signal Quality Monitoring Method. IEEE Sens. J..

[43] Wang J., Tang X., Ma P., Wu J., Ma C., Sun G. (2023). GNSS Spoofing Detection Using Q Channel Energy. Remote Sens..

[44] Yakkati R.R., Pardhasaradhi B., Zhou J., Cenkeramaddi L.R. A Machine Learning based GNSS Signal Classification. Proceedings of the 2022 IEEE International Symposium on Smart Electronic Systems (iSES).

[45] Zidan J., Adegoke E.I., Kampert E., Birrell S.A., Ford C.R., Higgins M.D. (2021). GNSS Vulnerabilities and Existing Solutions: A Review of the Literature. IEEE Access.

[46] Jafarnia-Jahromi A., Broumandan A., Nielsen J., Lachapelle G. (2012). GPS spoofer countermeasure effectiveness based on using signal strength noise power and *C*/*N*_0_ observables. Int. J. Satellite Commun. Netw..

[47] Zhu X., Lu Z., Hua T., Yang F., Tu G., Chen X. (2022). A Novel GPS Meaconing Spoofing Detection Technique Based on Improved Ratio Combined with Carrier-to-Noise Moving Variance. Electronics.

[48] Pardhasaradhi B., Yakkati R.R., Cenkeramaddi L.R. (2022). Machine Learning-Based Screening and Measurement to Measurement Association for Navigation in GNSS Spoofing Environment. IEEE Sens. J..

[49] Magiera J. (2019). A Multi-Antenna Scheme for Early Detection and Mitigation of Intermediate GNSS Spoofing. Sensors.

[50] Zhao Y., Shen F., Xu D., Meng Z. (2022). A Coprime Array-Based Technique for Spoofing Detection and DoA Estimation in GNSS. IEEE Sens. J..

[51] Chen J., Wang X., Fang Z., Jiang C., Gao M., Xu Y. (2024). A Real-Time Spoofing Detection Method Using Three Low-Cost Antennas in Satellite Navigation. Electronics.

[52] Yang H., Jin R., Xu W., Che L., Zhen W. (2023). Satellite Navigation Spoofing Interference Detection and Direction Finding Based on Array Antenna. Sensors.

[53] Meurer M., Konovaltsev A., Appel M., Cuntz M. Direction-of-Arrival Assisted Sequential Spoofing Detection and Mitigation. Proceedings of the 2016 International Technical Meeting of the Institute of Navigation.

[54] Liu R., Yang Z., Chen Q., Liao G., Zhu Q. (2023). Localization of GNSS Spoofing Interference Source Based on a Moving Array Antenna. Remote Sens..

[55] Zhang Z., Zhan X. (2018). Statistical analysis of spoofing detection based on TDOA. IEEJ Trans. Electr. Electron. Eng..

[56] Mao P., Yuan H., Chen X., Gong Y., Li S., Li R., Luo R., Zhao G., Fu C., Xu J. (2023). A GNSS Spoofing Detection and Direction-Finding Method Based on Low-Cost Commercial Board Components. Remote Sens..

[57] Chen S., Ni S., Lei T., Cheng L., Song X. (2024). GNSS Spoofing Detection via the Intersection Angle between Two Directions of Arrival in a Single Rotating Antenna. Sensors.

[58] Chang H., Pang C., Zhang L., Guo Z. (2022). Rotating Single-Antenna Spoofing Signal Detection Method Based on IPNN. Sensors.

[59] Xie J., Liu Q., Wang L., Gong Y., Zhang Z. (2022). Localizing GNSS Spoofing Attacks Using Direct Position Determination. IEEE Sens. J..

[60] Spravil J., Hemminghaus C., von Rechenberg M., Padilla E., Bauer J. (2023). Detecting Maritime GPS Spoofing Attacks Based on NMEA Sentence Integrity Monitoring. J. Mar. Sci. Eng..

[61] Xiao L., Li X., Wang G. GNSS Spoofing Detection Using Pseudo-range Double Differences between Two Receivers. Proceedings of the 2019 IEEE 7th International Conference on Computer Science and Network Technology (ICCSNT).

[62] Semanjski S., Muls A., Semanjski I., De Wilde W. Use and Validation of Supervised Machine Learning Approach for Detection of GNSS Signal Spoofing. Proceedings of the 2019 International Conference on Localization and GNSS (ICL-GNSS).

[63] Siemuri A., Selvan K., Kuusniemi H., Valisuo P., Elmusrati M.S. (2022). A Systematic Review of Machine Learning Techniques for GNSS Use Cases. IEEE Trans. Aerosp. Electron. Syst..

[64] Aissou G., Slimane H.O., Benouadah S., Kaabouch N. Tree-based Supervised Machine Learning Models for Detecting GPS Spoofing Attacks on UAS. Proceedings of the 2021 IEEE 12th Annual Ubiquitous Computing, Electronics & Mobile Communication Conference (UEMCON).

[65] Aissou G., Benouadah S., El Alami H., Kaabouch N. Instance-based Supervised Machine Learning Models for Detecting GPS Spoofing Attacks on UAS. Proceedings of the 2022 IEEE 12th Annual Computing and Communication Workshop and Conference (CCWC).

[66] Rossouw van der Merwe J., Nikolikj A., Kram S., Lukcin I., Nadzinski G., Rügamer A., Felber W. Blind Spoofing Detection for Multi-Antenna Snapshot Receivers using Machine-Learning Techniques. Proceedings of the 33rd International Technical Meeting of the Satellite Division of the Institute of Navigation (ION GNSS+2020).

[67] Rustamov A., Gogoi N., Minetto A., Dovis F. Assessment of the Vulnerability to Spoofing Attacks of GNSS Receivers Integrated in Consumer Devices. Proceedings of the 2020 International Conference on Localization and GNSS (ICL-GNSS).

[68] Liu J., Chen F., Xie Y., Ge B., Lu Z., Sun G. (2023). Robust Spoofing Detection for GNSS Array Instrumentation Based on *C*/*N*_0_ Difference Measurements. IEEE Trans. Instrum. Meas..

[69] Huang L., Yang Q. Low-cost GPS simulator GPS spoofing by SDR. Proceedings of the DEFCON.

[70] Rustamov A., Minetto A., Dovis F. (2023). Improving GNSS Spoofing Awareness in Smartphones via Statistical Processing of Raw Measurements. IEEE Open J. Commun. Soc..

[71] Ceccato S., Formaggio F., Caparra G., Laurenti N., Tomasin S. Exploiting side-information for resilient GNSS positioning in mobile phones. Proceedings of the 2018 IEEE/ION Position, Location and Navigation Symposium (PLANS).

[72] Guo C., Yang Z. Robust RF Fingerprint Extraction Scheme for GNSS Spoofing Detection. Proceedings of the 36th International Technical Meeting of the Satellite Division of the Institute of Navigation (ION GNSS+ 2023).

[73] Humphreys T.E., Bhatti J.A., Shepard D.P., Wesson K.D. The Texas Spoofing Test Battery: Toward a Standard for Evaluating GPS Signal Authentication Techniques. Proceedings of the 25th International Technical Meeting of the Satellite Division of the Institute of Navigation (ION GNSS 2012).

[74] Albright A., Powers S., Bonior J., Combs F. (2020). Oak Ridge Spoofing and Interference Test Battery (OAKBAT)—GPS.

[75] Foruhandeh M., Mohammed A.Z., Kildow G., Gerdes R., Berges R. (2020). SatGrid Dataset, Realtime Genuine and Spoofing Traces of GPS Signals Collected at Different Geographical Locations, Times and Environmental Conditions.

[76] Manfredini E.G., Akos D.M., Chen Y.-H., Lo S., Walter T., Enge P. Effective GPS Spoofing Detection Utilizing Metrics from Commercial Receivers. Proceedings of the 2018 International Technical Meeting of The Institute of Navigation.

[77] Spens N., Lee D.-K., Nedelkov F., Akos D. (2022). Detecting GNSS Jamming and Spoofing on Android Devices. NAVIGATION J. Inst. Navig. Sept..

[78] Spens N., Lee D.-K., Akos D. An application for detecting GNSS jamming and spoofing. Proceedings of the 34th International Technical Meeting of the Satellite Division of the Institute of Navigation (ION GNSS+).

[79] Zhang J., Cui X., Xu H., Lu M. (2019). A Two-Stage Interference Suppression Scheme Based on Antenna Array for GNSS Jamming and Spoofing. Sensors.

[80] Hu Y., Bian S., Li B., Zhou L. (2018). A Novel Array-Based Spoofing and Jamming Suppression Method for GNSS Receiver. IEEE Sens. J..

[81] Morales Ferre R., de la Fuente A., Lohan E.S. (2019). Jammer Classification in GNSS Bands via Machine Learning Algorithms. Sensors.

[82] Shafiee E., Mosavi M.R., Moazedi M. (2018). Detection of Spoofing Attack using Machine Learning based on Multi-Layer Neural Network in Single-Frequency GPS Receivers. J. Navig..

[83] Kartchner D.R., Palmer R., Jayaweera S.K. Satellite navigation anti-spoofing using deep learning on a receiver network. Proceedings of the 2021 IEEE Cognitive Communications for Aerospace Applications Workshop.

[84] Li W., Huang Z., Lang R., Qin H., Zhou K., Cao Y. (2016). A Real-Time Interference Monitoring Technique for GNSS Based on a Twin Support Vector Machine Method. Sensors.

[85] Wu Z., Zhao Y., Yin Z., Luo H. Jamming signals classification using convolutional neural network. Proceedings of the 2017 IEEE International Symposium on Signal Processing and Information Technology (ISSPIT).

[86] Alkhatib M., McCormick M., Williams L., leon A., Camerano L., Al Shamaileh K., Devabhaktuni V., Kaabouch N. Classification and Source Location Indication of Jamming Attacks Targeting UAVs via Multi-output Multiclass Machine Learning Modeling. Proceedings of the 2024 IEEE International Conference on Consumer Electronics (ICCE).

[87] Wu P., Calatrava H., Imbiriba T., Closas P. Jammer classification with Federated Learning. Proceedings of the 2023 IEEE/ION Position, Location and Navigation Symposium (PLANS).

[88] Swinney C.J., Woods J.C. GNSS Jamming Classification via CNN, Transfer Learning & the Novel Concatenation of Signal Representations. Proceedings of the 2021 International Conference on Cyber Situational Awareness, Data Analytics and Assessment (CyberSA).

[89] Miralles D., Levigne N., Akos D.M., Blanch J., Lo S. Android Raw GNSS Measurements as the New Anti-Spoofing and Anti-Jamming Solution. Proceedings of the 31st International Technical Meeting of the Satellite Division of the Institute of Navigation (ION GNSS+ 2018).

[90] Scott L. J911: The case for fast jammer detection and location using crowdsourcing approaches. Proceedings of the 24th International Technical Meeting of The Satellite Division of the Institute of Navigation (ION GNSS 2011).

[91] Axell E., Eklöf F.M., Johansson P., Alexandersson M., Akos D.M. (2015). Jamming Detection in GNSS Receivers: Performance Evaluation of Field Trials. J. Inst. Navig..

[92] Strizic L., Akos D.M., Lo S. Crowdsourcing GNSS Jammer Detection and Localization. In Proceedings of the 2018 International Technical Meeting of the Institute of Navigation.

[93] Olsson G.K., Axell E., Larsson E.G., Papadimitratos P. Participatory Sensing for Localization of a GNSS Jammer. Proceedings of the 2022 International Conference on Localization and GNSS (ICL-GNSS).

[94] Olsson G.K., Nilsson S., Axell E., Larsson E.G., Papadimitratos P. Using Mobile Phones for Participatory Detection and Localization of a GNSS Jammer. Proceedings of the 2023 IEEE/ION Position, Location and Navigation Symposium (PLANS).

[95] Yozevitch R., Marbel R., Flysher N., Ben-Moshe B. (2021). Save Our Roads from GNSS Jamming: A Crowdsource Framework for Threat Evaluation. Sensors.

[96] Osman A., Moussa M.M.E., Tamazin M., Korenberg M.J., Noureldin A. (2020). DOA Elevation and Azimuth Angles Estimation of GPS Jamming Signals Using Fast Orthogonal Search. IEEE Trans. Aerosp. Electron. Syst..

[97] Morales Ferre R., Richter P., De La Fuente A., Lohan E.S. In-lab validation of jammer detection and direction finding algorithms for GNSS. Proceedings of the 2019 International Conference on Localization and GNSS (ICL-GNSS).

[98] Thanakan K., Sapphaniran K., Palasarn T., Supnithi P., Phakphisut W., Sakorn C. Real-Time Jamming Detection and Position Estimation via Software-Defined Radio (SDR). Proceedings of the 2021 18th International Conference on Electrical Engineering/Electronics, Computer, Telecommunications and Information Technology (ECTI-CON).

[99] Medina D., Lass C., Marcos E.P., Ziebold R., Closas P., García J. On GNSS Jamming Threat from the Maritime Navigation Perspective. Proceedings of the 2019 22th International Conference on Information Fusion (FUSION).

[100] Panice G., Luongo S., Gigante G., Pascarella D., Di Benedetto C., Vozella A., Pescape A. A SVM-based detection approach for GPS spoofing attacks to UAV. Proceedings of the 2017 23rd International Conference on Automation and Computing (ICAC).

[101] Tohidi S., Mosavi M.R. Effective detection of GNSS spoofing attack using a multi-layer perceptron neural network classifier trained by PSO. Proceedings of the 2020 25th International Computer Conference, Computer Society of Iran (CSICC).

